# Sources of heterogeneity in human monocyte subsets

**DOI:** 10.1016/j.imlet.2013.03.004

**Published:** 2013-04

**Authors:** Laura J. Appleby, Norman Nausch, Nicholas Midzi, Takafira Mduluza, Judith E. Allen, Francisca Mutapi

**Affiliations:** a Institute for Immunology & Infection Research, Centre for Immunity, Infection & Evolution, School of Biological Sciences, University of Edinburgh, EH9 3JT, UK; b National Institutes of Health Research, P.O. Box CY 573, Causeway, Harare, Zimbabwe; c Department of Biochemistry, University of Zimbabwe, P.O. Box 167, Mount Pleasant, Harare, Zimbabwe

**Keywords:** LPS, lipopolysaccharide, MHC, major histocompatibility complex, PBMC, peripheral blood mononuclear cell, LN2, liquid nitrogen, MFI, mean fluorescence index, FSC, forward scatter, SSC, side scatter, NK, natural killer, ELISA, enzyme linked immunosorbent assay, SWAP, soluble worm antigen preparation, ANOVA, analysis of variance, DP, double positive, SEM, standard error of mean, Monocyte, Human, Phenotype, CD14, CD16

## Abstract

Human monocytes are commonly defined and discriminated by the extent of their cell surface expression of CD14 and CD16, with associated differences in function and phenotype related to the intensity of expression of these markers. With increasing interest into the function and behaviour of monocytes, it is important to have a clear understanding of how differing strategies of analysis can affect results and how different protocols and population backgrounds can affect this highly morphogenic cell type.

Using PBMCs from populations with differing ethnicities and histories of parasite exposure we have characterized monocyte phenotype based on intensity of CD14 and CD16 expression. Using the surface markers HLA-DR, CCR2 and CX3CR1, we compared monocyte phenotype between populations and further assessed changes in monocytes with freezing and thawing of PBMCs.

Our results reveal that there is a progression of surface marker expression based on intensity of CD14 or CD16 expression, stressing the importance of careful gating of monocyte subtypes. Freezing and thawing of the PBMCs has no effect generally on the monocytes, although it does lead to a decrease in CD16 and CX3CR1 expression. We show that there are differences in the monocyte populations based on ethnicity and history of exposure to the common parasites *Plasmodium falciparum* and *Schistosoma haematobium.*

This study highlights that blood monocytes consist of a continuous population of cells, within which the dominant phenotype may vary dependent on the background of the study population. Comparing results from monocyte studies therefore needs to be done with great care, as ethnic background of donor population, gating strategy and processing of PBMCs may all have an effect on outcome of monocyte phenotype.

## Highlights

• Variation in monocyte phenotype is explored using the markers CD14, CD16, HLA-DR, CX3CR1 and CCR2.
• The CD14++CD16+ monocytes exhibit a spectrum of markers dependent on location within the gate.
• Monocyte phenotype varies dependent on genetic background and history of exposure to infection.
• Processing technique for PBMC purification does not lead to changes in monocyte subsets.
• Processing technique for purification can alter intensity but not pattern of marker expression.

## 1 Introduction

Peripheral blood monocytes, which represent around 10% of circulating leukocytes in humans, are recognized as the largest pool of circulating progenitor cells and form a vital part of the immune system [1,2]. The enormous heterogeneity in human monocyte size, morphology, phagocytic function and cell adhesion was first described in 1989 [3] and was quickly followed by multiple attempts to discriminate monocyte subtypes. Recently new nomenclature was suggested by an expert panel in Brescia, Italy to define three subsets according to expression of CD14 and CD16 [4]. The major subset consists of CD14highCD16negative monocytes (CD14++CD16−), while the CD16 expressing monocytes are usually divided into a CD14highCD16low (CD14++CD16+) and a CD14lowCD16high (CD14+CD16++) subset. These groupings can identify monocytes that differ in surface expression of chemokine markers, major histocompatibility complex (MHC) class II expression and in their capacity to produce cytokines and phagocytose microbial particles [1,5–8]. However, while there have been some in-depth genetic and proteomic analyses of monocyte functions and cell markers [1,6,9], there is still no universally accepted demarcation of these subsets based on phenotypic markers [9]. Furthermore, there is no visible clustering of the cell subsets based on the CD14 and CD16 surface markers, instead the two markers form a spectrum of expression levels potentially contributing to variation between experiments [4,7,10]. Differential expression of chemokine and scavenger receptors indicates a functional potential in terms of trafficking to sites of infection and inflammation. Indeed, monocyte migration and trafficking has been observed to vary between subsets based on expression of CCR2 and CX3CR1 [11]. Another feature of monocytes is their ubiquitous expression of the MHC class II surface receptor, HLA-DR, which is frequently used to distinguish between CD16 expressing monocytes and CD16 expressing NK cells [12]. As a receptor that is involved in antigen presentation [9], it is often considered an activation marker [13–15] and indicates functional differences for the monocyte subsets as well as subset activation status [12].

Thus far, the majority of human monocyte studies have taken place using volunteers of Caucasian background and in high income countries where pathologies arising from non-communicable diseases such as atherosclerosis, liver cirrhosis and asthma dominate [16]. This means that, despite the demonstration of the importance of monocytes in experimental models of parasitic diseases [2,17,18], comparatively little is known about the nature, phenotype and development of monocytes in people exposed to tropical infectious diseases. Furthermore the majority of studies investigating monocyte phenotype and function use whole blood or fresh PBMCs rather than cryopreserved peripheral blood mononuclear cells (PBMCs). Cryopreservation of PBMCs is an indispensable tool for longitudinal clinical studies as well as during fieldwork when samples have to be stored and transported from the collection point to a laboratory. Furthermore, the capability to retrospectively analyze specimens from the same patient allows analysis of large sample populations, monitoring of clinical status over time or after treatment and improves accuracy while reducing within-patient as well as interassay variability [19,20]. To date, studies determining the effects of cryopreservation on PBMCs have focused on cell compartment changes [21] or maturation pathways [22], but no studies have been conducted on the effects of cryopreservation on the cell phenotype which is central to the function of the monocytes.

In this study our aims were (1) to determine changes in expression levels of cell surface markers occurring within the monocyte cell population dependent on CD14 and CD16 expression intensity, (2) to assess the stability of these markers during processes involved in freezing and storage, and finally, (3) to determine if differences occur in the proportion and phenotype of monocytes in the different sub-populations between Caucasian individuals who have been exposed to a typical western lifestyle, and African individuals who are lifelong residents of a rural helminth endemic area.

## 2 Methods

### 2.1 Ethical statement

Written consent was obtained from all participants or their guardians prior to enrolling in the study. Local ethical approval was given and local review board guidelines adhered to. The cohort of rural Africans was part of a larger study investigating the epidemiology and immunology of human schistosomiasis that was conducted in the Mashonaland East Province of Zimbabwe. Permission to conduct the study in the region was obtained from the Provincial Medical Director. Institutional and ethical approval was received from the University of Zimbabwe and the Medical Research Council of Zimbabwe respectively. At the beginning of the study, parents and guardians of participating children had the aims and procedures of the project explained fully in the local language, Shona, and written consent was obtained from participants’ parents/guardian before enrolment into the study. After collection of all samples, all participants and their parents/guardians were offered anthelmintic treatment with the recommended dose of praziquantel (40 mg/kg of body weight).

### 2.2 Study populations

To address the different questions, three different cohorts were used, which are described in Table 1. For the purposes of phenotyping monocytes for cell surface expression patterns of the markers CCR2, CX3CR1 and HLA-DR, a cohort of 62 individuals living in a rural area where *Schistosoma haematobium* is prevalent was used. All participants were from the Murehwa district in north eastern Zimbabwe. All individuals recruited into each study were *S. haematobium* and co-infection negative and had never received anti-helminthic treatment. In addition there is little or no infection with *Schistosoma mansoni*, soil transmitted helminths and malaria transmission is sporadic and seasonal [23]. The residents of the area are subsistence farmers with frequent contact with infected water for purposes of irrigation, bathing, washing and collecting water (assessed by questionnaire) [24].

In order to investigate the effects of cryopreservation on monocyte phenotype and cell numbers, peripheral blood from nine African or Caucasian volunteers, currently living in urban environments, was used to compare monocytes from freshly isolated PBMCs to those from cryopreserved PBMCs. For evaluation of differences that genetics and lifetime exposure to infection may have on monocyte phenotype, PBMCs from 21 Africans who were exposed to, but negative for, helminth, malaria and HIV co-infections were compared to 21 age and sex matched Caucasians with no reported exposure to these pathogens. Table 2 shows the ages (mean, median and range) of each of the populations (rural African and Caucasian) used in background comparisons. In order to check for effects of genetic background vs pathogen exposure, five donors of African origin (Kenya (*n* = 4) and Zimbabwe (*n* = 1)) were recruited to the study. All five donors had grown up in an urban environment.

### 2.3 Parasitology

Parasite infection status was determined in the Zimbabwean participants through examination of at least 2 stool and 2 urine samples collected on 3 consecutive days and a single blood sample. The urine samples were used for microscopic diagnosis of *S. haematobium* infection using the standard urine filtration method [25], while the stool sample was used for microscopic diagnosis of intestinal helminths and *S. mansoni* infection using the Kato–Katz method [26]. Blood smears were microscopically examined for *Plasmodium falciparum* infection, and results confirmed using the rapid Paracheck test, (Orchid Biomedical Systems, Goa, India) and serologically tested for HIV status using the DoubleCheckGold™ HIV1&2 test kit (Orgenics, Ltd., Yavne Israel). All Zimbabwean donors were selected to be *S. haematobium*, *S. mansoni*, soil transmitted helminth, malaria and HIV negative.

### 2.4 Blood collection and isolation of PBMC

Approximately 30 ml of venous blood was collected in heparinised tubes from all donors. PBMC were isolated through density centrifugation using Lymphoprep™ (Axis-Shield, Cambridgeshire, UK). Heparinised plasma was collected and stored at −80 °C until assay. PBMCs were counted and resuspended at approximately 1 × 10^7^ c/ml in freezing media (90%DMSO, 10%FCS) for cryopreservation and immediately cooled to −80 °C in a freezing container (Nalgene Nunc, International) prior to placing in liquid nitrogen (LN2) until assay. For analysis of fresh PBMCs a further 8 ml of venous blood was collected from each individual on the day of thawing and processing cryopreserved samples. The time between processing PBMCs for cryopreservation and processing PBMCs for a fresh analysis was no more than a month in any case. Processing was performed in the manner described and cells were suspended at 5 × 10^6^ cells/ml. Surface staining was performed straight away in the same manner as for cryopreserved cells as described.

### 2.5 Phenotyping of monocytes

Cryopreserved PBMCs were thawed straight from LN2 in a 37 °C water bath until only a small crystal remained. The contents of the vial were slowly added to complete media (RPMI 1640), supplemented with 10% heat inactivated FCS, 2 mM l-glutamine and 100 U penicillin/streptomycin (all Lonza, Verviers, Belgium). Cells were washed twice with complete media, counted in trypan blue (Sigma–Aldrich, Dorset, UK). Cells were washed in PBS (Lonza, Verviers, Belgium) and resuspended at 5 × 10^5^ cells per stain for each staining panel.

Fresh and thawed cells for staining were incubated with 10% FCS at 4 °C for 10 minutes prior to staining and stained with Alexa488-conjugated anti-CD14 (clone M5E2), PE-Cy7-conjugated HLA-DR (clone L243; all from BD Biosciences, San Jose, CA), Pacific Blue-conjugated CD16 (clone CB16; eBiosciences, San Diego, CA), Alexa647-conjugated CX3CR1 (clone 2A9-1; BioLegend, San Diego, CA), PerCP-conjugated CCR2 (clone 48607; RnD Systems, Minneapolis, MN) or the relevant isotype control for 30 min at 4 °C. Unbound antibodies were washed off and cells were resuspended in PBS prior to acquisition of at least 50,000 live events on a BD FACS LSR II (BD Biosciences, San Jose, CA). Compensation was performed prior to acquisition of each experiment using BD FacsComp beads (BD Biosciences, San Jose, CA). Analysis was performed using FlowJo software (TreeStar, USA) and Mean fluorescence index (MFI) was calculated for each marker with the relevant isotype control subtracted.

### 2.6 Monocyte discrimination

To ensure that only CD14+ cells representing monocytes were analyzed, a gating strategy was employed to gate only HLA-DR, CD14 expressing cells. Briefly a live gate, to include all leukocytes, was drawn based on forward scatter (FSC) and side scatter (SSC). HLA-DR positive cells were gated to exclude any CD16+ natural killer (NK) cells and other non-MHC expressing cells [12], and true monocytes were gated based on expression of CD14 and CD16 surface markers.

### 2.7 Determination of exposure to *P. falciparum* and *S. haematobium*

In order to determine if any arising differences between the Caucasian and African participants were due to undetected schistosome or *Plasmodium* parasite infection (current or previous) or parasite-unrelated mechanisms such as genetic differences, serological assays were conducted to determine parasite exposure history. Enzyme linked immunosorbent assay (ELISA) was used to measure antigen-specific antibodies to malaria schizont (IgG and IgM) and schistosome adult worm (IgG4, IgM, IgE) in the serum. Lyophilized soluble *S. haematobium* adult worms (SWAP) was obtained from the Theodor Bilharz Institute (Giza, Egypt) and reconstituted as recommended by the manufacturer. Schizont extract was a kind gift from David Cavanagh (University of Edinburgh, UK). ELISAs were performed as reported elsewhere [27,28], and all ELISAs were performed in duplicate on the same day with positive and negative controls on each plate.

### 2.8 Statistical analysis

All statistical analyses were conducted using the statistical package SPSS version 19. Parametric tests were used when assumptions of parametric tests were met, otherwise non-parametric tests were used [29]. When using parametric tests data were transformed using appropriate transformations: surface marker expression (measured as MFI) was log transformed (log_10_(*x* + 1)), proportions of subsets were arcsine square root transformed, and antibody responses were square root transformed. In parametric models age was taken as a continuous variable, sex (male/female) and donor ethnicity (African/Caucasian) were categorical.

To test the hypothesis that the whole monocyte population is composed of a continuum of ‘subsets’ consisting of distinct phenotypic profiles, differences in expression of surface markers were analyzed using a one way analysis of variance (ANOVA) with subset as a grouping variable and post hoc tests used to determine significant differences between adjacent subsets. Differences in the proportion of each subset were analyzed using an arcsine square root transformation and a one way ANOVA using type I sequential sums of squares in a similar manner as discussed. When sample size and assumptions did not allow, the Kruskal–Wallis test was used to test for differences between surface marker expression and subset proportion.

In order to investigate the effects of cryopreservation on monocyte phenotype with respect to changes in proportions of subsets and expression of phenotypic markers, the Wilcoxon Signed Ranks Test was used with processing method (fresh PBMCs vs. cryopreserved PBMCs) as grouping variable. Differences in intensity of surface marker expression between subsets were determined by MANOVA, allowing for sex and age using type I sequential sums of squares.

The effects of exposure history to parasitic infection on monocyte subsets were tested by ANOVA, allowing for sex (categorical) and age (continuous) using type I sequential sums of squares. Differences in positive antibody responses to parasite antigen between populations were tested using the Chi-squared test for association after categorizing responses into positive (OD > 0) or negative (OD = 0). Significant *p* values are reported as *p* ≤ 0.05 unless otherwise indicated.

## 3 Results

### 3.1 Discrimination of MHC II positive monocytes

**Fig. 1:**
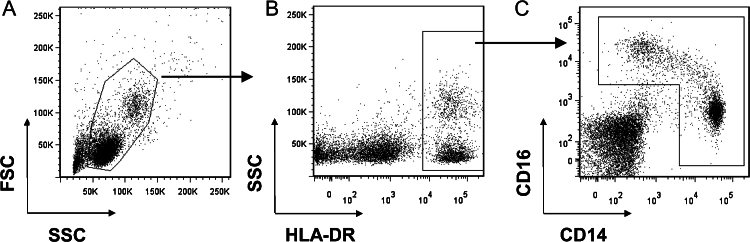
Representative flow cytometry dot plots demonstrating the gating strategy employed to define CD14+ monocytes. (A) Live gate for total leukocytes based on the forward scatter (FSC) and side scatter (SSC) properties, (B) separation of monocytes from non HLA-DR expressing CD14 expressing NK cells prior to (C) gating total monocytes based on CD14 and CD16 expression.

Monocytes represent a population of MHCII cells that express varying levels of both CD14 and CD16 surface markers. Fig. 1 illustrates the gating strategy used that excludes non-MHCII, CD16 positive NK cells, but includes HLA-DR and CD14 positive monocytes. The commonly observed ‘banana’ shape that is seen with this cell population and the lack of clustering within the double positive population is demonstrated in Fig. 1C.

### 3.2 Different gating strategies give different phenotypic profile patterns

**Fig. 2:**
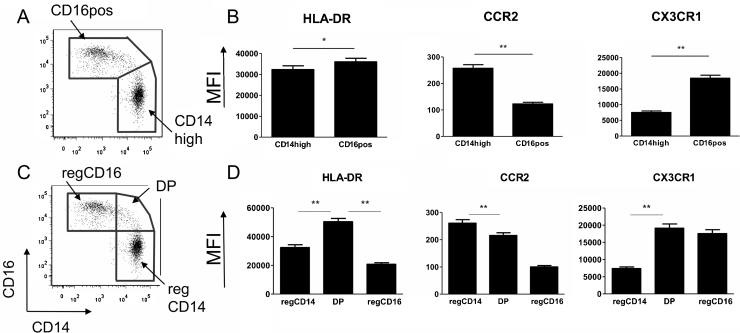
Examples of previously published gating strategies using the same representative donor as in Fig. 1. Top panel demonstrating (A) the two gating strategy based on CD16 positive and CD16 negative monocytes, and (B) the phenotypic profile associated with the two subsets. Lower panel demonstrates (C) the gating strategy of three subsets, regCD14, DP and regCD16 based on CD14 and CD16 expression, and (D) the associated surface marker expression profile. Significant *p* values are from a post hoc one-way ANOVA. Significant differences are indicated with * (*p* < 0.05) or ** (*p* < 0.001).

**Fig. 3:**
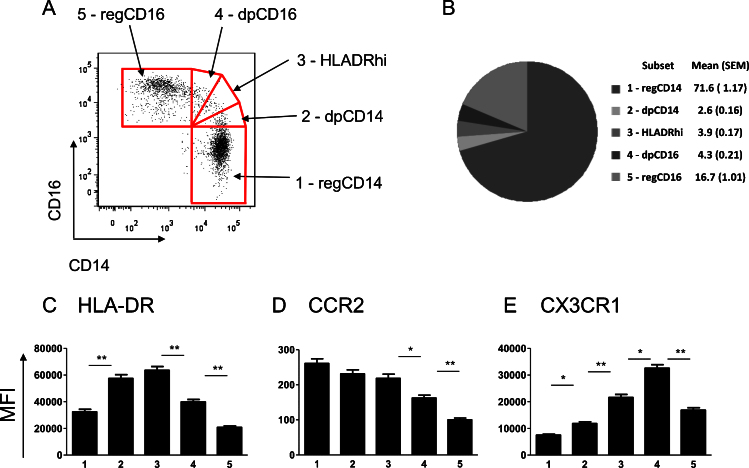
Division of monocyte population into five subsets with varying levels of expression of CD14 and CD16. (A) Representative dot plots demonstrating the division of the five subsets to include the traditional CD14++CD16− (regCD14), CD14+CD16++ (regCD16) and three subsets within the CD14 CD16 double positive population: dpCD14, HLADRhi, dpCD16. (B) Pie chart illustrating the proportions, mean and (SEM) of the five subsets seen in the rural African population (*n* = 62). (C)–(E) Mean MFI for the rural African population of different monocyte phenotypic surface markers within each of the five subsets: 1: regCD14; 2: dpCD14; 3: HLADRhi; 4: dpCD16; 5: regCD16. (C) HLA-DR, (D) CCR2 (E) CX3CR1. Significant *p* values are from a post hoc one-way ANOVA. Significant differences are indicated with * (*p* < 0.05) or ** (*p* < 0.001).

There is currently no consensus on the best gating strategy of monocytes with at least three different methods published that involve not only different markers of definition [4,30,31] but also different numbers of subsets [32–34]. Thus we investigated if there were differences in the basic phenotypic characteristics of monocytes based on different gating strategies according to CD14 and CD16 MFI. We found stark differences in subset expression of surface markers while employing different gating strategies as demonstrated in Fig. 2. In sub-setting the monocytes into two groups (Fig. 2 upper panels) there is an obvious difference in patterns of expression in comparison to three groupings (Fig. 2, lower panels). Expression of the phenotypic markers CX3CR1 and HLA-DR is observed to be higher in the middle double positive (DP) (CD14++CD16+) monocytes than in the CD16 monocytes; a difference which is lost in the two subset strategy. Due to a lack of clarification in the literature about the discrimination of the DP monocytes, we were interested in determining if the DP expressing subpopulation of monocytes was a subset phenotypically distinct from both the CD14++CD16− and the CD14+CD16++ monocyte populations. Based on expression of HLA-DR [12], we divided this middle population into three separate subsets to give a double positive CD14high population (dpCD14), a double positive CD16high population (dpCD16) and a double positive HLA-DR high population (HLADRhi) (Fig. 3A). The HLADRhi designation was based on this group expressing the highest levels of HLA-DR (Fig. 3C). Gating in such a manner would identify any differences occurring both within this group, as well as between this group and the adjacent subsets. For the purposes of this manuscript we wished to maintain a distinction between what has been previously published and agreed to (three subsets defined as CD14++CD16−, CD14++CD16+ and CD14+CD16++) and the monocyte groupings as we defined them here. We thus decided to designate the CD14++CD16− as regCD14 monocytes and the CD14+CD16++ as regCD16 monocytes. The gating of all five subsets is shown in Fig. 3A with the proportions of each of the subsets illustrated in Fig. 3B.

### 3.3 Rather than distinct subsets, the monocyte gate consists a spectrum of progressively changing phenotypic markers

From the larger cohort of 62 Africans we analyzed the five monocyte groupings with respect to changes in their surface expression of the phenotypic markers CCR2 and CX3CR1, and the MHC receptor HLA-DR. Fig. 3C–E shows the mean expression levels of these markers in each of the monocyte groupings. The significantly elevated level of HLA-DR in the HLADRhi subset compared to dpCD16 (*p* < 0.001) and the regCD14 cells (*p* < 0.001) may indicate an increased activation status in these cells (Fig. 3C). CCR2 shows a spectrum of expression levels with the highest on the regCD14 monocytes, decreasing with increasing CD16 expression (Fig. 3D). The CD14high expressing monocytes (regCD14, dpCD14 and HLADRhi) do not show significant differences in CCR2 expression, however with increasing CD16 expression (transitioning the subset from regCD14 towards regCD16) there is a concurrent decrease in CCR2 expression (from HLADRhi to dpCD16: MFI difference = −55.48, *p* = 0.001, and from dpCD16 to regCD16 MFI difference = −63.68, *p* < 0.001). In contrast, CX3CR1 shows significant differences in expression level across all subsets (Fig. 3E), with the lowest expression of CX3CR1 on CD14++ monocytes (regCD14 and dpCD14) as previously reported [35]. Interestingly, the highest expression is in the dpCD16 monocytes with a significant decrease in expression between dpCD16 and regCD16 monocytes (dpCD16 to regCD16 MFI difference = −14,414, *p* < 0.001) (Fig. 3E). The regCD16 monocytes show a significant decrease in marker expression compared to dpCD16 for all analyzed markers (Fig. 3C–E).

### 3.4 Total monocyte number but not subset proportion differs between fresh and cryopreserved PBMCs

**Fig. 4:**
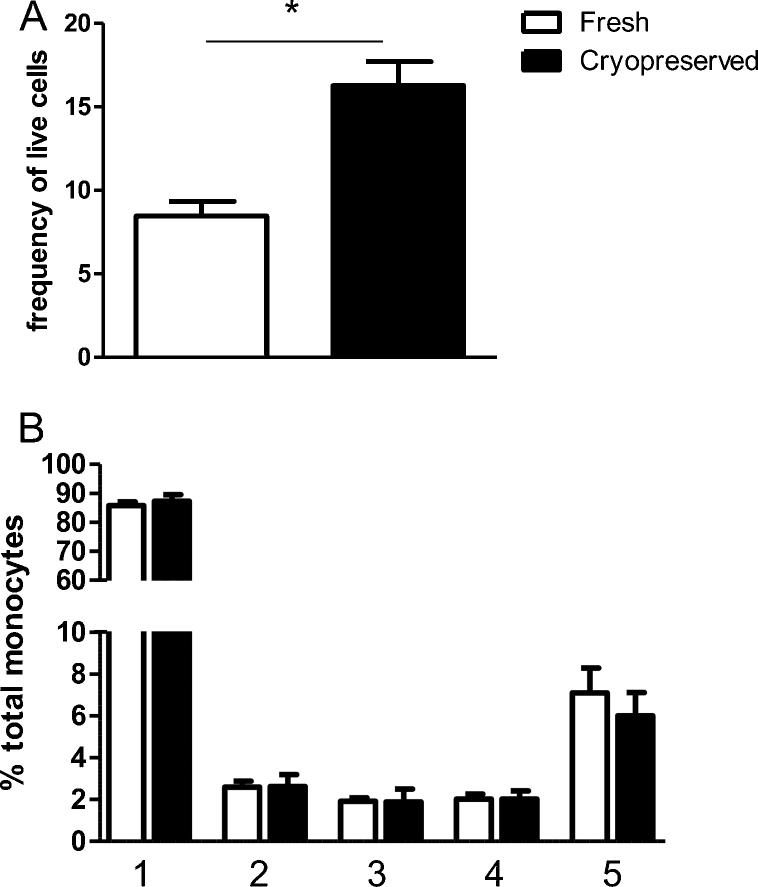
Differences between fresh and cryopreserved PBMCs from 9 individuals with an urban background (African: *n* = 5; Caucasian: *n* = 4) were compared. (A) Fresh and cryopreserved monocytes as frequency of live gated population. Monocytes from fresh PBMCs show a significantly smaller proportions of monocytes in comparison to cryopreserved PBMCs, measured non-parametrically with Wilcoxon Signed Ranks Test (*z* = −2.67, *p* = 0.004). (B) Mean and SEM of proportions of the five subsets in fresh and cryopreserved preparations. 1: regCD14; 2: dpCD14; 3: HLADRhi; 4: dpCD16; 5: regCD16. Open bars: fresh PBMCs; closed bars: cryopreserved PBMCs. There are no significant differences in proportions dependant on preparation method (measured non-parametrically using the Wilcoxon signed ranks test).

**Fig. 5:**
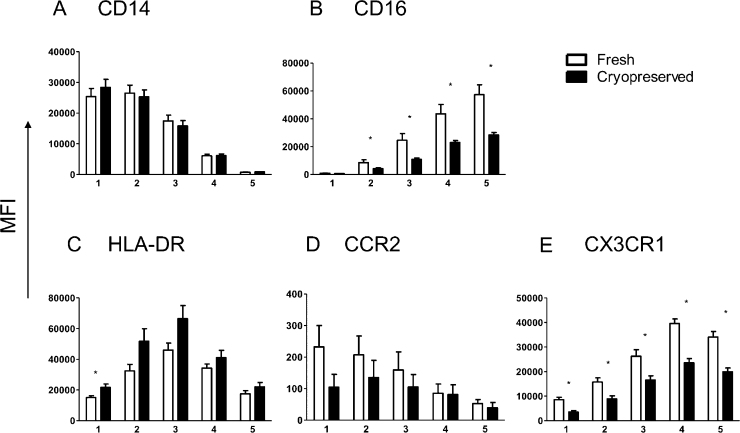
Differences observed in cell surface phenotype between fresh (open bar, *n* = 9) and cryopreserved (closed bar, *n* = 9) preparations of cells. (A) CD14, (B) CD16, (C) HLA-DR, (D) CCR2 and (E) CX3CR1. 1: regCD14; 2: dpCD14; 3: HLADRhi; 4: dpCD16; 5: regCD16. Significant differences (*p* < 0.05) are from nonparametric Wilcoxon signed rank test and are indicated with *.

To investigate whether monocytes change their expression levels and phenotype dependent on cryopreservation, nine donors of Caucasian or African descent, with predominantly urban backgrounds, had a collection of peripheral blood for PBMC purification and cryopreservation. In a second blood draw, fresh PBMCs were purified, and these were stained on the same day as their cryopreserved cells. Cryopreserved cells show a greater proportion of monocytes as a percentage of live gated cells, as shown in Fig. 4A (*z* = −2.67, *p* = 0.004). However there were no significant differences in the proportion of subsets as tested by one-way ANOVA and repeated measures as shown in Fig. 4B. Differences in cell surface expression between fresh and cryopreserved monocytes are indicated in Fig. 5. No differences are seen in CD14, HLA-DR or CCR2 expression (Fig. 5A, C and D), but significant differences are seen in almost all subsets for CD16 and CX3CR1 surface expression (Fig. 5B and E, respectively), with fresh PBMCs showing a higher MFI for all subsets in both markers.

### 3.5 African and Caucasian donors show differences in proportions of monocyte subsets

**Fig. 6:**
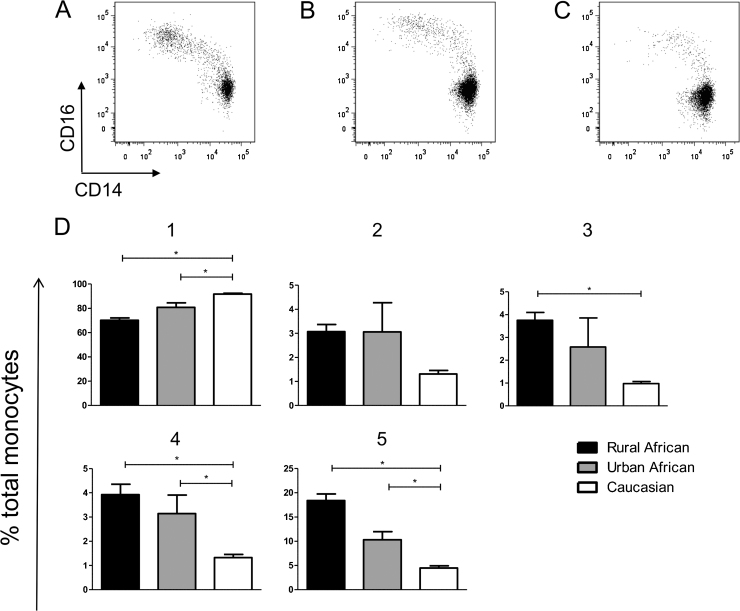
Top Panel (A)–(C) representative CD14/CD16 dot plots of monocytes from (A) a rural African donor, (B) an urban African donor and (C) a Caucasian donor. Cells are gated according to the strategy described in Fig. 1. Bottom panel (D) Bar graphs illustrating the differences in mean and SEM of monocyte subset proportions between rural Africans (*n* = 21, filled bars), Africans from urban environments (*n* = 5, grey bars) and Caucasians (*n* = 21, open bars). 1: regCD14; 2: dpCD14; 3: HLADRhi; 4: dpCD16; 5: regCD16. *P* values are from the Mann–Whitney test with an applied Bonferonni correction. Significant *p* values (*p* < 0.0167) are indicated with *.

**Fig. 7:**
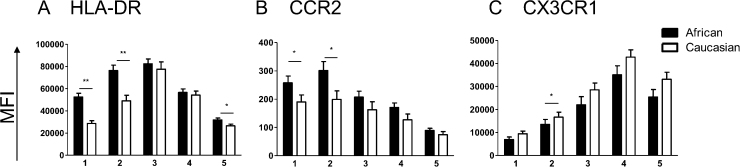
Mean expression levels of surface markers between rural Africans (closed bars, *n* = 21) and Caucasians (open bars, *n* = 21) based on surface marker by subset. (A) HLA-DR, (B) CCR2, (C) CX3CR1. 1: regCD14; 2: dpCD14; 3: HLADRhi; 4: dpCD16; 5: regCD16. Significant *p* values from Mann Whitney test are represented by * (*p* ≤ 0.05) and ** (*p* ≤ 0.001).

As the majority of studies investigating monocytes have taken place in high income areas, we were interested in whether the phenotypic patterns we have characterized were a feature of the study population or if they can be transferred across populations. We therefore undertook an investigation to phenotype monocytes from donors of different ethnicities. Fig. 6A–C illustrates the differences seen in flow analysis between (A) rural African monocytes (B) urban African monocytes and (C) Caucasian monocytes. There is a stark difference in the proportion of CD16 and CD14 expressing subsets between the ethnicities. Fig. 6D shows the proportions of the five subsets in the whole African and Caucasian populations that we sampled. Caucasians are exhibiting a significantly greater proportion of regCD14 cells compared to rural Africans (Fig. 6D, 1), while monocytes from Africans have a significantly greater proportion of all other subsets except dpCD14 (Fig. 6D, 2–5). Fig. 7 shows the differences in expression levels of surface markers between these subsets for the rural African and Caucasian groups. Interestingly, while the surface marker MFIs between the two populations are not always similar, they follow the same pattern of expression for all subsets. The surface expression of the activation marker HLA-DR is higher on the rural African population, predominantly in the CD14high monocytes (Fig. 7A). Similarly, CCR2 is significantly higher on rural African monocytes in the regCD14 and dpCD14 groups (Fig. 7B). The expression of the chemokine and adhesion receptor CX3CR1 is higher in the Caucasian cohort, although this is only significant in the dpCD14 subset (Fig. 7C).

### 3.6 Caucasians and rural Africans demonstrate different histories of parasite exposure

**Fig. 8:**
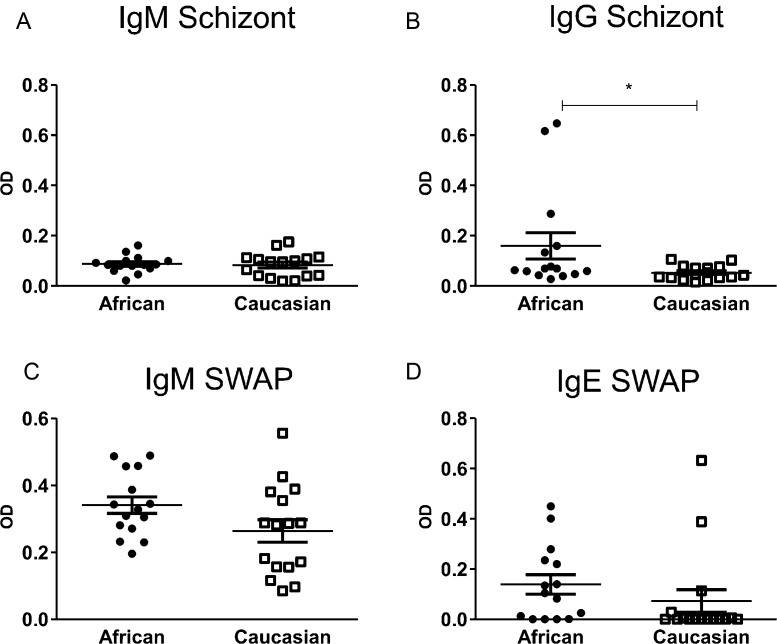
Antibody responses of rural Africans (closed circles, *n* = 15) and Caucasians (open squares, *n* = 16) to parasite antigens. (A) IgM and (B) IgG response to malaria schizont. (C) IgM and (D) IgE responses to *S. haematobium* adult worms (SWAP) as measured by ELISA. Significant *p*-values are from Type I sequential sums of squares and are indicated with * (*p* < 0.05).

In order to understand whether the observed differences in subset proportions between the different populations are due to history of exposure to parasitic exposure, we were interested in antibody responses to both *P. falciparum* and *S. haematobium* antigens. Fig. 8 shows the antibody responses to *P. falciparum* schizont antigens (upper panels A and B) and SWAP (lower panels C and D). There was very little IgM antibody response to malaria schizont in either the rural African or Caucasian populations, indicating that neither group had recent exposure to the parasite (Fig. 8A). In contrast, the rural Africans had a greater IgG response to the schizont antigen in comparison to the Caucasians (*F* = 8.042, *p* = 0.009), as evidence of previous exposure (Fig. 8B) [36,37]. IgM against SWAP is associated with recent exposure to schistosomiasis, and both populations showed low levels of SWAP specific IgM, although the rural African population shows slightly elevated levels in comparison to the Caucasians (Fig. 8C) (mean OD = 0.579, Standard error of mean (SEM) = 0.021 and mean OD = 0.497, SEM = 0.033 respectively). As both groups are negative for schistosomiasis defined by egg count in urine, the low IgM response is not surprising. IgE against SWAP is associated with cumulative history of exposure to the parasite antigen. Fig. 8D shows that there is no significant difference between the rural African and Caucasian populations with regards to IgE responses (*F* = 3.684, *p* = 0.066). However the number of IgE positive responders to SWAP in the African group is significantly greater than the number of IgE positive responders to SWAP in the Caucasian group (80% and 37.5% positive responders respectively; *χ*^2^ (2) = 5.743, *p* = 0.017). Taken together this indicates that while neither population is demonstrating current exposure to either plasmodium or schistosome parasite infection, the rural Africans have had more history of exposure to the schistosome adult worm and are showing signs of developing immunity to the parasite.

## 4 Discussion

In humans, the three identified monocyte subsets have differing migration, maturation and functional potential [4,35], and there have been reports of an increase in the CD14+CD16++ subset in numerous pathologies [5,8,38–40], however the definition of CD14+CD16++ monocytes varies within each of these studies. By dividing the monocytes into five separate subsets, we have demonstrated that the CD14++CD16+ subset is made up of a phenotype with significant variation in the expression levels of typical markers. The decision to subdivide what is commonly known as the CD14++CD16+ subset was based on inconsistency within the field as to where this division lay, combined with the observation that HLA-DR is expressed most highly by what we defined as the HLADRhi subset [9,12]. As HLA-DR is an activation marker [41,42] this may indicate a functional role for these monocytes that is not shared by either of the dpCD14 or dpCD16 monocytes. This method of division of the middle CD14+CD16++ monocyte population gives a clear indication that there is a progressive pathway between the monocyte subsets. This continuum may not be surprising considering recent murine data demonstrating that the Ly6C+ monocytes (correlate of human CD14++CD16+ and CD14++CD16− monocytes) are precursors of Ly6C− monocytes (correlate of human CD14+CD16++) [43].

CCR2 and CX3CR1 are chemokine receptors that have frequently been reported to have disparate affiliations with monocyte subsets [3,35]. Here we report no significant differences in CCR2 expression within CD14++ expressing subsets, with significant differences in expression only occurring with the onset of CD16 expression. In contrast CX3CR1 expression shows a clear distinction in expression levels within each of the five subsets and differential expression between the dpCD16 and regCD16 monocytes. As CX3CR1 is involved in adhesion to the blood vessel wall and with rapid extravasation of the cell [11], this may be indicative of a separate function for the regCD16 (CX3CR1low) monocytes and the dpCD16 (CX3CR1high) monocytes. Indeed the regCD16 subset consistently shows significant decreases in surface marker expression to the dpCD16 subset.

Due to time or physical restrictions, as well as for longitudinal cohort studies, many research protocols including our own require cryopreservation of PBMCs before processing and staining for flow cytometry. Therefore we investigated whether there were changes in these defined five subsets based on processing of the cells. We show that total monocyte numbers were reduced following cryopreservation. However, there were no significant differences in the proportions of each of the subsets. The MFI of CD14 and CD16 were examined, in particular as the MFI of CD16 has been reported to be upregulated with activation [44,45]. The CD16 receptor was expressed to a higher intensity on fresh than cryopreserved cells. Similarly CX3CR1 showed a significantly higher MFI in each subset for the fresh PMBCs. These differences may be a consequence of the freezing process, perhaps influencing the stability of these markers and preventing a rapid activation of the thawed cells in the same manner as the fresh preparation. Previous studies have found no differences in frozen and fresh PBMCs with regards to T cell proportion and function [46] as well as macrophage differentiation [22]. Here we have found that while the MFI between the two preparations can alter, the patterns remain robust, indicating that while the two methods are comparable they should not be used to see within experiment differences.

For any study evaluating interventions that may alter blood monocytes, it is important to know if genetic and environmental background differences between individuals impact on monocyte subset balance. We show here that such differences do exist in Africans and Caucasians, with Africans exhibiting a greater proportion of CD16 expressing subsets compared to Caucasians. All individuals were negative for helminth infections, malaria and HIV. However, the rural African population did show evidence of past exposure to malaria and schistosomiasis infection. In many pathologies frequently occurring in urban environments, such as asthma, microbial infection and arthritis, there is a reported increase in the CD14++CD16+ and CD14+CD16++ subsets, which may be indicative of an activated immune system [8,38,39]. Knowledge of these baseline subset differences is important for undertaking studies in environments where exposure to numerous pathogens is common, as changes seen with other infections may not be as distinct. Interestingly, the pattern of monocyte subsets in the African group living in a western environment lay between the Caucasians and the rural Africans exposed to schistosomiasis. This indicates that the differences seen in the subset patterns may be more of a function of exposure to parasites than genetics. However, this group of individuals was small, and only one was originally from Zimbabwe, where the rural Africans originated from. There is reportedly more human genetic diversity within Africa than in the rest of the world so these differences could be as much to do with genetic differences between the populations as with the exposure history [47]. In terms of differences in phenotype of the monocyte subsets, similar to the preparations of the cells, the pattern of expression of the subset markers remained the same between the different ethnicities. Previous reports have shown that CCR2 is low to negative on CD16 expressing monocytes [31], whereas our group, in studying PBMCs isolated from individuals in Zimbabwe, has always found evidence of a certain expression level of this marker (unpublished data). We show here that the PBMCs from rural Africans express CCR2 to a greater intensity in comparison to Caucasians, particularly in the CD14 expressing monocytes. Overall, expression of HLA-DR was higher in the rural African population than in Caucasians. As an activation marker that is rapidly upregulated with infection, the HLA-DR expression level may be indicating recent exposure to infection or, perhaps an impaired ability to shed HLA-DR into the serum in response to inflammation [13,14,48]. CX3CR1 showed a tendency towards higher expression in Caucasian monocytes similar to CD16 expression (data not shown) and is in agreement with the differences in CD16 observed with preparation differences. Previously reported associations between CD16 and CX3CR1, as well as with CD14 and CCR2, make it unsurprising that the significant differences are seen in the same direction between these markers within these populations. Taken together it is clear that while monocyte subset markers do not change pattern between different populations, the expression levels, as well as proportions of subsets can be significantly different. This may be due to a lifetime of exposure to pathogens, such as malaria and schistosomiasis, and the immune response associated with this exposure. Whilst the sample size reported on is small, the findings do highlight the importance of taking care when comparing results from different experiments, or in recruiting individuals into a study. Further research into differences in monocyte subsets based on ethnic background would be valuable in fully understanding the extent of these differences.

## 5 Conclusions

In this study we present data demonstrating the spectrum of maker expression within the recently defined subsets of human monocytes [4]. With direct relevance to research in the field we show that there are few changes in these subsets or expression of surface markers in response to cryopreservation. We also show that expression levels of typical markers for monocyte function do change in intensity based on ethnic background of the individual. While the scope of this study does not allow for determining what drives these changes, it does emphasize the important role monocytes have in exposure to disease.

Our study focused on surface marker expression, but it will be interesting to assess differences in intracellular markers, cytokine secretion and particularly functional capabilities within these five subsets. Whilst sorting the cells in large enough quantities for functional analysis may be a challenge, using this monocyte gating technique we have shown that there is a shifting spectrum of phenotypic markers that may lead to clues as to the function of each of the monocyte subsets. Importantly our study indicates that conformity across research groups in gating of these subsets is necessary in order to compare studies.

## Funding

This work was supported by the World Health Organization and the Wellcome Trust (grant number WT082028MA), the Thrasher Foundation and from the Medical Research Council (grant number LJA-544 to [LJA] and G0600818 to [JEA]).

## Figures and Tables

**Table 1:** Study cohorts and description.

Study	Donor ethnicity	Origin (urban/rural)	*N*
Whole monocyte phenotype	African (Zimbabwe)	Rural	62
Effects of cryopreservation on monocyte phenotype	African (other)	Urban	5
	Caucasian		4
Effects of genetics or exposure on monocyte phenotype	Europe	Urban	21
	African (Zimbabwe)	Rural	21

**Table 2:** Age of rural African and Caucasian cohorts.

	Rural African				Caucasian			
	*N*	Mean	Median	Range (min–max)	*N*	Mean	Median	Range (min–max)
M	6	23	17	15–55	10	33.6	31	26–53
F	15	31.1	34	17–44	11	33	29	25–54
Total	21	28.8	28	15–55	21	33.3	30	25–54
